# *Biological Procedures Online *now publishing with BioMed Central

**DOI:** 10.1186/1480-9222-13-2

**Published:** 2011-01-31

**Authors:** Shulin Li

**Affiliations:** 1 The University of Texas MD Anderson Cancer Center, Houston, TX 77030, USA

## 

We are excited to inform all previous and future contributors, as well as readers, that *Biological Procedures Online *has been transferred to the open access publisher BioMed Central, a powerhouse for the timely publishing of articles in online journals.

I am honored to continue to act as Editor-in-Chief, serving alongside editorial board members from various well-renowned institutions. In joining BioMed Central, we are confident that the journal will have a much stronger impact in the near future. Based on the many high quality publications published in 2009 and 2010, it is our belief that our impact factor will make a strong recovery next year.

*Biological Procedures Online *is an open access, peer-reviewed journal which publishes articles that improve access to techniques and methods in the medical and biological sciences. Articles feature step-by-step protocols that allow researchers to implement methods in "cookbook" fashion at the bench top.

*Biological Procedures Online *is interested in any small or large technical innovation in the field of biology, with an emphasis on biomedical science. Our special interests include: technological innovation related to stem cells, siRNA, genomics, genome sequencing, nanomedicine, drug delivery and experimental protocols. We welcome research articles, case reports, reviews, commentaries, methodology articles and short reports.

By continuing to publish as an open access journal, all articles published in *Biological Procedures Online *will be freely available for anyone to view. *Biological Procedures Online *defrays the cost of the publication process via article-processing charges because it does not have subscription charges for its content. This policy allows open access journals to reach a much larger audience than subscription-based journals [1], with the possibility of higher impact factors as a result [2,3]. The journal's articles are archived in PubMed Central [4], complying with a number of funding bodies including the NIH, Wellcome Trust, and Howard Hughes Medical Institute [5-8]. *Biological Procedures Online *is also indexed by a number of prominent services, including Thomson Reuters (ISI), BIOSIS, CABI, CAS, EmBase, Scopus and the Zoological Record.

All manuscripts submitted to *Biological Procedures Online *will be subjected to a quick and impartial closed peer-review process. The Editor-in-Chief/assigned editors will initially examine the manuscripts to check the animal assurance and proper handling of human tissues. Based on a review of the results, and the recommendations from the assigned editors, the Editor-in-Chief will inform authors regarding acceptance of their manuscripts. Upon acceptance, the articles will appear online immediately and be posted on PubMed soon after.

We are delighted to be taking *Biological Procedures Online *forward with BioMed Central and look forward to receiving research articles, methodology articles and protocols for consideration.

## References

[B1] Suber P Open access, impact, and demand BMJ 2005 330 1097 10.1136/bmj.330.7500.1097 15891208 PMC557876

[B2] Hitchcock The effect of open access and downloads ('hits') on citation impact: a bibliography of studies http://opcit.eprints.org/oacitation-biblio.html

[B3] Brody T Harnad S Earlier Web Usage Statistics as Predictors of Later Citation Impact http://eprints.ecs.soton.ac.uk/10713/02/timcorr.htm

[B4] PubMed Central http://www.pubmedcentral.org

[B5] Which funding agencies explicitly allow direct use of their grants to cover article processing charges? http://www.biomedcentral.com/info/about/apcfaq#grants

[B6] NIH Calls on Scientists to Speed Public Release of Research Publications http://www.nih.gov/news/pr/feb2005/od-03.htm

[B7] Wellcome Trust position statement in support of open and unrestricted access to published research http://www.wellcome.ac.uk/node3302.html

[B8] Howard Hughes Medical Institute Research Policies - Public access publishing http://www.hhmi.org/about/research/sc320.pdf

